# Knowledge of community members on COVID-19 in Ibadan, Oyo State, Nigeria

**DOI:** 10.11604/pamj.2021.39.17.24715

**Published:** 2021-05-06

**Authors:** Olayinka Stephen Ilesanmi, Aanuoluwapo Adeyimika Afolabi

**Affiliations:** 1 Department of Community Medicine, College of Medicine, University of Ibadan, Oyo State, Nigeria; 2 Department of Community Medicine, University College Hospital, Ibadan, Oyo State, Nigeria

**Keywords:** Knowledge, COVID-19, Nigeria, health education, coronavirus

## Abstract

**Introduction:**

more cases of COVID-19 continue to be reported in Nigeria. The level of knowledge could prompt individuals to take precautionary measures and reduce the spread. This study aimed to assess the knowledge of community members in Ibadan on COVID-19.

**Methods:**

using a descriptive cross-sectional study design, an interviewer-administered semi-structured questionnaire was used to obtain information from adult community members. Multistage sampling methods were used to select respondents from four local government areas (LGAs). Knowledge scores of causes, mode of spread, signs and symptoms and preventive measures were computed. Scores above the mean were categorized as satisfactory. Bivariate chi-square tests and binary logistic regression were performed on respondents' characteristics and knowledge of COVID-19.

**Results:**

respondents interviewed were 480 with a median age of 32 years (range: 18-80 years), and 191 (39.8%) aged between 25 and 34 years. Females were 275 (57.3%), 18 (3.8%) had never heard of COVID-19. The main source of information was radio 88.1% and television 54.3%. In all the knowledge domains 200 (43.3%) had satisfactory knowledge of COVID-19. Respondents in Ibadan North-West LGA had five times odds [OR=5.23 (95%CI=2.87-9.54)] of having satisfactory knowledge of COVID-19 while respondents in Ibadan North-East LGA had three times odds [OR=3.35 (95%CI=1.85-6.09)] compared to those in Ibadan South East LGA.

**Conclusion:**

an even dissemination strategy of COVID-19 information across the communities is required. More sensitization and health education sessions should be broadcast on the radio and television. Regular COVID-19 broadcast is required to improve the knowledge level of community members.

## Introduction

The novel coronavirus disease (COVID-19, SARS-CoV-2, or nCov-2) is an emerging respiratory illness which was first detected in Chinese Wuhan City [1]. It was declared a global pandemic by the World Health Organization on the 11^th^ March, 2020 [1]. Currently, COVID-19 has been transmitted to more than 200 countries with nearly 8 million confirmed cases and 440,000 deaths globally [2]. Presently, the infection has taken its toll on Africa with over 200,000 confirmed cases and more than 6000 deaths as of 19^th^ June, 2020 [2]. As a part of containment measures, many countries including Nigeria have commenced knowledge improvement strategies for their residents regarding COVID-19 [3].

The index case of COVID-19 in Nigeria was detected on the 27^th^ February, 2020 [3]. As at mid-June, 2020, Oyo State of Nigeria has recorded a total of 764 confirmed cases of COVID-19, and ranks fourth in the country [4,5]. As a part of the mitigation efforts to prevent further transmission, the Nigerian government commenced improved public health campaigns regarding COVID-19. The Nigerian government established the emergency operations centre in collaboration with partner agencies, and its activities have been led by experts [6]. Included in the response activity is the targeting of community members especially individuals who live in denial of the existence of the coronavirus [5]. The success of these interventions is depends on the knowledge level of public behavior which stems from individual´s knowledge [7].

Improvement of community awareness has been proposed along with other public health measures as effective means for reducing community transmission of infections [8,9]. Lessons from the 2003 SARS outbreak suggest that the knowledge is associated with the level of panic and emotions, and this could complicate or enhance containment or control attempts regarding the disease [10]. Evidence from literatures on Ebola virus disease have reported the effectiveness of knowledge in modelling preventive attitudes and practices [11]. Similarly, studies on COVID-19 in China have demonstrated that adequate knowledge of the infection could prompt individuals to take precautionary measures [12].

It is currently unknown if community members in Ibadan possess satisfactory knowledge regarding COVID-19 outbreak, its means of transmission, signs and symptoms and preventive measures. Adequate knowledge of community members is likely to contribute to prompt containment of COVID-19 [10]. It is therefore necessary to determine the level of knowledge of these individuals. This study thus aimed at assessing the knowledge of community members in Ibadan regarding COVID-19.

## Methods

**Study area:** the study was carried out in Ibadan, Oyo State Nigeria. Ibadan is the capital city of Oyo State. By mid-June, a total of 764 confirmed cases of COVID-19 has been reported in Oyo State and the state ranked fourth on the total number of cases of COVID-19 in Nigeria [4,5]. The official language in Nigeria is English, while the major informal language for communication in Ibadan is Yoruba, which has different dialects.

**Study population:** the study population for the survey was one eligible member of the households in the selected communities in Ibadan, Oyo State. Consenting household members were included in the study. Household members that were less than 18 years were excluded.

**Study design:** a descriptive cross-sectional study design was used. Data was collected using an interviewer administer questionnaire. Data collection took place from the 3^rd^ June to the 6^th^ June 2020.

**Sample size determination:** the sample size was calculated using sample size formula for descriptive cross-sectional study. A prevalence of 50% was used due to non-availability of a study to use for the sample size calculation. We calculated a sample size of 427.

**Sampling technique:** a multistage sampling technique was used to select the respondents for the study. Stage 1: simple random sampling was used to select 4 out of the 11 local government area in Ibadan. Stage 2: in each of the selected LGA, a political ward was chosen out of the 11 political wards. Stage 3: a center location was chosen in the selected ward. A bottle was rotated to determine the direction of movement of the interviewers. From the direction of the bottle tip all consenting eligible adults from the households were included in the study until 120 persons were interviewed in each LGA. Sampling of 120 each in the four selected LGA gives a total sample size of 480.

**Study instrument:** the questionnaire had two sections. Section A: sociodemographic characteristics. Section B: knowledge of the respondents on COVID-19.

**Data collection methods and instruments:** data were collected using a semi-structured interviewer-administered questionnaire. Data collection was done by trained research assistants with minimum of first degree. The questionnaire was pretested among adult residents of a LGA that was not selected for the study (Ibadan South-West).

**Data management:** data were analyzed with SPSS version 23. Chi square test was used for the assessment of significant associations between proportions. Age was summarized using mean and standard deviation, while frequencies, and percentages were used for categorical variables. Knowledge scores were computed with "+1" assigned for correct response and “0” assigned for incorrect response. Using the mean score as the cut-off point, these scores were graded as satisfactory or not satisfactory knowledge. Bivariate chi-square test and multivariate logistic analyses were performed on respondents´ characteristics and knowledge of COVID-19. Variables in the bivariate test with p value of < 0.05 were accepted as significant.

**Ethical considerations:** ethical approval to carry out the study was obtained from the Oyo State Ministry of Health Ethical Review Committee, with reference number AD/13/479/1779A. Permission for the study was sought from the respondents and their confidentiality was ensured. The respondents were informed of their right to decline or withdraw from the study at any time without any adverse consequences. No harm came to participants because of participation in this study.

## Results

**Figure 1:**
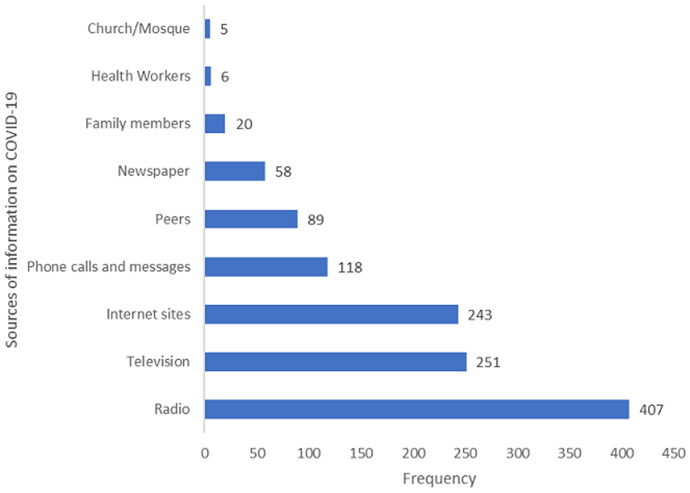
sources of COVID-19 information among Ibadan residents, 2020

A total number of 480 respondents were interviewed with median age of 32 years (range: 18-80 years), 191 (39.8%) aged between 25 and 34 years. Females were 275 (57.3%), Christians were 281 (58.5%), those with secondary education and above were 415 (86.5%) while 224 (46.7%) of the respondents engaged in business or trading (Table 1). Among the respondents, 462 (96.3%) persons have heard of COVID-19, their main sources of information were radio 407 (88.1%) and television 251 (54.3%). Other sources of information on COVID-19 were as shown in Figure 1. Table 2 shows the respondents with satisfactory score on COVID-19 across all domains. In Ibadan North-East LGA, the overall knowledge was satisfactory among 54 (50%) of the respondents. The overall knowledge in Ibadan South-East LGA was satisfactory among 26 (22.6%) respondents. Across all the LGAs, 200 (43.3%) had satisfactory knowledge of COVID-19 in all the domains. Among respondents aged less than 25 years, 45 (46.9%) had satisfactory knowledge compared to 22 (40%) of those 45 years and above, among females, 121 (45.7%) had satisfactory knowledge compared to males 79 (40.1%), though the differences are not significant statistically. Among the respondents with secondary education and above, 180 (45.1%) had satisfactory knowledge compared to 20 (31.7%) with primary education and below (p<0.05). In all, 24 (64.9%) professionals and civil servants had satisfactory knowledge compared to 77 (36%) of traders (p=0.001). Satisfactory knowledge among residents in Ibadan North-West LGA was 72 (60.5%), while it was 26 (22.6%) in Ibadan South-East LGA (p<0.001). Other associations between sociodemographic characteristics and knowledge of COVID-19 are shown in Table 3. Table 4 shows the determinants of satisfactory knowledge of COVID-19 among Ibadan residents. Respondents in Ibadan North-West LGA had five times odds [OR=5.23(95%CI=2.87-9.54)] of having satisfactory knowledge of COVID-19 while respondents in Ibadan North-East LGA had three times odds [OR=3.35(95%CI=1.85-6.09)] compared to those in Ibadan South East LGA.

**Table 1:** socio-demographic characteristics of respondents among Ibadan residents, 2020

Socio-demographic characteristics	Frequency	%
**Age group (years)**		
<25	98	20.4
25-34	191	39.8
35-44	135	28.1
≥45	56	11.7
**Sex**		
Male	205	42.7
Female	275	57.3
**Religion**		
Christianity	281	58.5
Islam	199	41.5
**Highest level of education**		
Primary and below	65	13.5
Secondary and above	415	86.5
**Ethnicity**		
Yoruba	427	89.0
Ibo	38	7.9
Hausa	8	1.7
Others	7	1.5
**Occupation**		
Business/trader	224	46.7
Artisans	159	33.1
Professional/civil servant	38	7.9
Unemployed/housewife/student	59	12.3

**Table 2:** respondents with satisfactory score on COVID-19 across all domains among Ibadan residents, 2020

LGA	Causes n (%)	Mode of spread n (%)	Signs and symptoms n (%)	Preventive measures n (%)	Overall knowledge n (%)
Ibadan North-East (n=108)	16 (14.8)	88 (81.5)	82 (75.9)	73 (67.6)	54 (50)
Ibadan South-East (n=115)	0 (0)	91 (79.1)	39 (33.9)	58 (50.4)	26 (22.6)
Ibadan North-West (n=119)	80 (67.2)	82 (68.9)	97 (81.5)	66 (55.6)	72 (60.5)
Egbeda (n=120)	81 (67.5)	69 (57.5)	74 (61.7)	58 (48.3)	48 (40.0)

**Table 3:** association between sociodemographic characteristics and satisfactory knowledge of COVID-19 among Ibadan residents, 2020

Variable	Satisfactory knowledge n (%)	Unsatisfactory knowledge n (%)	Chi-square	p-value
**Age group (Years)**				
<25	45 (46.9)	51 (55.3)	0.781	0.854
25-34	79 (42.7)	106 (57.3)		
35-44	54 (42.9)	72 (57.1)		
≥45	22 (40)	33 (60)		
**Sex**				
Male	79 (40.1)	118 (59.9)	1.422	0.233
Female	121 (45.7)	144 (54.3)		
**Religion**				
Christianity	122 (44.9)	150 (55.1)	0.658	0.417
Islam	78 (41.1)	112 (58.9)		
**Highest level of education**				
Primary and below	20 (31.7)	43 (68.3)	3.960	0.047
Secondary and above	180 (45.1)	219 (54.9)		
**Ethnicity**				
Yoruba	171 (41.6)	240 (58.4)	4.487	0.213
Ibo	21 (58.3)	15 (41.7)		
Hausa	4 (50)	4 (50)		
Others	4 (57.1)	3 (42.9)		
**Occupation**				
Business/trader	77 (36)	137 (64)	15.436	0.001
Artisans	77 (50.3)	76 (49.7)		
Professional/civil servant	24 (64.9)	13 (35.1)		
Unemployed/housewife/student	22 (37.9)	36 (62.1)		
**Location**				
Ibadan North-East	54 (50)	54 (50)	36.910	< 0.001
Ibadan South-East	26 (22.6)	89 (77.4)		
Ibadan North-West	72 (60.5)	47 (39.5)		
Egbeda	48 (40)	72 (60)		

**Table 4:** determinants of satisfactory knowledge of COVID-19 among Ibadan residents, 2020

Sociodemographic characteristics	B	Odds ratio	95% CL for OR		p-value
			Lower	Upper	
Primary school education and below		1			
Secondary education and above	0.294	1.342	0.723	2.491	0.351
Business/trader	-0.387	0.679	0.358	1.290	0.237
Artisans	0.085	1.088	0.558	2.123	0.804
Professional/civil servant	0.867	2.379	0.964	5.873	0.060
Unemployed/housewife/student		1			
Ibadan South-East LGA		1			
Ibadan North-East LGA	1.210	3.352	1.846	6.088	0.001
Ibadan North-West LGA	1.655	5.232	2.870	9.539	0.001
Egbeda LGA	0.877	2.403	1.314	4.394	0.004

## Discussion

This study found that 96.3% of the respondents have heard about COVID-19. Among those who have heard, 43.3% who had satisfactory knowledge level were recorded. This implies that hearing alone does not translate to satisfactory knowledge of COVID-19. The low level of knowledge obtained from this study is not consistent with similar studies conducted in China, Egypt, and Iran, and an online cross-sectional study conducted in Nigeria [10,13-15]. Online studies are usually sent to those with internet access, who are usually more knowledgeable and biased. We also found no positive association between educational qualification and satisfactory knowledge of COVID-19. This observation is contrary to the influence of education on COVID-19 knowledge possessed by Chinese respondents [10]. Findings obtained from this study is thus worrisome. This is because knowledge is a prerequisite in the disease perception and uptake of preventive measures and practices regarding COVID-19.

Findings from this study explain that knowledge of COVID-19 is location-specific. Respondents in Ibadan North-East and North-West LGAs displayed satisfactory knowledge of COVID-19 compared to their counterparts in other LGAs. The novelty of this finding could be due to the location in which the study was conducted. An Ebolavirus study conducted in Lagos, Nigeria reported no association between residence in selected LGAs and satisfactory knowledge [11]. Also, a Chinese study reported contrary findings on the association between location and knowledge of COVID-19 [8,10]. This finding could help identify communities with unsatisfactory knowledge of COVID-19 to focus health education interventions among them.

Multivariate analysis conducted in the present study revealed that there is no significant association between occupation and satisfactory knowledge of COVID-19. This explains that respondents´ occupations had no influence on knowledge regarding COVID-19. An Ethiopian study conducted among Jimma University Medical Centre (JUMC) visitors similarly reported no association between respondents´ occupation and knowledge of COVID-19 [16]. However, health workers have been reported to possess adequate knowledge regarding COVID-19 compared to other groups in another study [17]. The findings from this study could influence and intensify public health campaigns regarding COVID-19 evenly across all professions.

Concerning the sources of information for COVID-19 among respondents, our results explain that traditional forms (radio and television) accounted for the main sources of knowledge concerning COVID-19. More novel channels such as internet sites (Facebook, Twitter, and WhatsApp) accounted for half of COVID-19 information gotten by respondents. The dominance of traditional media as sources of COVID-19 information was similarly reported in a Nigerian study [15]. Contrary to this finding, studies in Egypt revealed that Facebook is the main information site for COVID-19 [13]. Similarly, knowledgeable persons regarding Ebola virus in the United States identified the internet as the primary source of information [18]. In the present study, less than 10% have heard about COVID-19 from health workers and family members. Health workers are therefore expected to use the advantage of seeing different cadre of people to educate them. Also, collaborations have been put in place with both traditional and novel sources for the timely dissemination of COVID-19 information [3,6]. These channels present an accessible means for information sourcing and knowledge seeking, but if left unchecked could also spread false information regarding COVID-19. They could be flagbearers for rumors on what the COVID-19 illness is. Thus, checks to be placed on the use of these platforms to encourage the spread of only evidence-based information regarding COVID-19 [19]. These findings promote the need to focus COVID-19 information on traditional and novel sources of information with high coverage.

## Conclusion

Knowledge is key to prevention. The possession of satisfactory knowledge among community members is key to imbibing positive attitudes and practices regarding COVID-19. We hereby recommend an even dissemination strategy of COVID-19 information across all groups. Strategies for the communication of COVID-19 information to contain the pandemic need to be set up and implemented. Also, sensitization and health education sessions should be encouraged by family members and health workers to enhance adequate knowledge regarding COVID-19. Regular monitoring of broadcast on radio, television, and internet sites is required to ensure accurate reporting of COVID-19 information to improve the knowledge level of community members regarding the pandemic.

### What is known about this topic

- Lessons from the 2003 SARS outbreak suggest that the knowledge is associated with the level of panic and emotions and could complicate or enhance containment or control attempts regarding the disease;
- Evidence from literatures on Ebola virus disease have reported the effectiveness of knowledge in modelling preventive attitudes and practices;
- Studies on COVID-19 in China have demonstrated that adequate knowledge of the infection could prompt individuals to take precautionary measures.

### What this study adds

- To the best of our knowledge, this study is the first community-based survey conducted in Ibadan, Nigeria regarding the knowledge of COVID-19;
- We observed gaps in the knowledge of COVID-19 among community members. Traditional media was mostly the source of COVID-19 information;
- We propose that COVID-19 information be evenly disseminated and monitored across all groups and media, respectively.
